# ANALYSING PERFORMANCE ASSESSMENT IN PUBLIC SERVICES: HOW USEFUL IS THE CONCEPT OF A PERFORMANCE REGIME?

**DOI:** 10.1111/padm.12206

**Published:** 2015-09-02

**Authors:** STEVE MARTIN, SANDRA NUTLEY, JAMES DOWNE, CLIVE GRACE

**Affiliations:** ^1^ Steve Martin, James Downe and Clive Grace are in Cardiff Business SchoolCardiff UniversityUK; ^2^ Sandra Nutley is in the School of ManagementUniversity of St AndrewsUK

## Abstract

Approaches to performance assessment have been described as ‘performance regimes’, but there has been little analysis of what is meant by this concept and whether it has any real value. We draw on four perspectives on regimes – ‘institutions and instruments’, ‘risk regulation regimes’, ‘internal logics and effects’ and ‘analytics of government’ – to explore how the concept of a multi‐dimensional regime can be applied to performance assessment in public services. We conclude that the concept is valuable. It helps to frame comparative and longitudinal analyses of approaches to performance assessment and draws attention to the ways in which public service performance regimes operate at different levels, how they change over time and what drives their development. Areas for future research include analysis of the impacts of performance regimes and interactions between their visible features (such as inspections, performance indicators and star ratings) and the veiled rationalities which underpin them.

## INTRODUCTION

How closely, in what ways and by whom the performance of public sector organizations should be assessed is a topic that has been hotly debated by scholars of public administration as well as policy‐makers and practitioners (Van Dooren *et al.* 2010). In recent years, these questions have been accompanied by a growing concern about how to improve public service performance. A range of approaches to performance assessment have been adopted at different times, in different services and across different jurisdictions, and during the past decade a number of scholars have described these as ‘performance regimes’ (Roper *et al.* 2005; Grubnic and Woods 2009; Moynihan 2009; Moynihan *et al.* 2011; Charbonneau and Bellavance 2012). However, they have used the concept of a ‘regime’ in a general sense rather than as an analytical tool. What constitutes a performance regime – its distinguishing features and dimensions – has not been entirely clear.

The concept of a regime has been employed in several fields, including international relations, international law, urban policy and political science, to analyse governance processes at international, national and local levels, and regime theory has been applied to particular aspects of governance, including policy problems (Mays and Jochim 2013) and risk regulation (Hood *et al.* 2001). A common thread running through the application of the concept of a regime in these different contexts is that it encourages an analytical approach which explores the interactions among institutions and interest groups and ways in which these are mediated by formal and informal rules and cultures. This has the potential to support both comparative and longitudinal studies of governance arrangements. Studies often focus on visible interactions and espoused rationalities, but there is also a more reflexive form of regime theory – the analytics of government approach – which seeks to uncover the often hidden or invisible rationalities that sit behind particular modes of governance (Dean 1999).

On the face of it, then, employing the concept of regimes to analyse approaches to performance assessments in public sector settings should be revealing. However, there have been very few comparative analyses of performance assessment and no attempts to apply the concept of a regime to this task. As a result, there is an important gap in our knowledge about the value of the concept in this domain. This article seeks to address the gap by examining how the concept can be developed to advance understanding of the origins and operation of approaches to performance assessment. It does this empirically and analytically by exploring how four perspectives on regimes can help us to understand three recent approaches to performance assessment in UK local government.

The article addresses four specific questions. First, is a regimes perspective a useful way of identifying and analysing differences between approaches to performance assessment in public services? Second, at what level of analysis is the concept of a performance regime most useful? Third, can it help to explain differences in approaches to performance assessment between jurisdictions and over time? Finally, how might the concept of a performance regime be developed in the future to advance our understanding of approaches to performance assessment? In answering these questions, the article makes an important contribution to investigating the applicability of the concept of a regime to the study of performance assessment.

The next section focuses on four approaches to regime analysis which are particularly relevant to the study of performance assessment in the public sector. Following this, we explain our methods and data sources. Then we apply the concept of a performance regime to analyse approaches to the assessment of local government performance in England, Scotland and Wales. We describe the main characteristics of the approaches, the interactions between the actors who developed them and the instruments that they used. We conclude by examining our four research questions in light of this empirical analysis, reflecting on the implications of our research for the application of the concept of performance regimes to the study of public service organizations and suggesting areas for future research.

## REGIME THEORY AND PERFORMANCE REGIMES

All of the literatures in which the concept has been adopted identify a regime as consisting of interactions among actors (variously described as interest groups, coalitions and institutions) who operate within relatively stable systems characterized by formal and informal rules and cultures (e.g. Krasner 1982). Regimes can be forced upon actors by external circumstances, but while government actors are usually regarded as the principal architects of regimes, they need not necessarily be mandated or imposed by a hierarchical state (Bebbington *et al.* 2012).

Debates about the usefulness of a regimes perspective in international relations and urban studies (e.g. Strange 1982) provide insights into some of the potential benefits and limitations of applying the concept to the study of performance assessments of public service organizations. There are, however, four specific contributions that are particularly relevant for our development and application of the concept: Talbot's (2010) depiction of the institutions and instruments that make up a performance regime; the framework developed by Hood *et al.* (2001) to analyse approaches to risk regulation; the analysis by Pollitt *et al.* (2010) of the impact of exogenous shocks and internal logics on performance indicator sets; and Dean's (1999) analytics of government framework, which draws attention to the importance of invisible rationalities that shape regimes of practices.

Talbot was one of the first scholars to apply the term ‘regime’ to approaches to performance management in the public sector (Talbot *et al.* 2005; Talbot 2010). He argues that performance is shaped and steered by interactions among a multitude of institutions and instruments. Institutions which can play a role in regulating the performance of public service organizations include the legislature, executive, judicial and quasi‐judicial bodies; audit, inspection and regulatory bodies; professional institutes; partner organizations; user organizations; and service providers. Talbot (2010) suggests that, individually and collectively, these actors have access to a range of instruments, and illustrates this by reference to four strategies that were deployed by the Blair government in the UK in an attempt to improve public service performance: managerial–contractual instruments (performance reporting, contracts, targets and national minimum standards); markets and quasi‐markets (competitive tendering, benchmarking and performance league tables); pressure from service users; and interventions designed to enhance capability and capacity (for example, capability reviews, leadership programmes, changes in human resource practices and structural reorganizations).

Hood *et al.*'s (2001) analysis is particularly useful for our study because it makes the concept of a regime tractable, and the dimensions which it identifies connect with debates about performance assessment. They argue that risk regulation regimes consist of sets of interacting or interrelated parts which form relatively bounded systems that have a degree of continuity over time and can be specified at different levels of breadth or generality. They analyse regimes in terms of three features: the ‘context’ within which they operate; their ‘content’ (institutional configurations); and the ‘control mechanisms’ that they deploy. According to Hood *et al.* (2001), examining these three cross‐cutting dimensions of a regime makes it possible to capture ‘the complex of institutional geography, rules, practice, and animating ideas that are associated with the regulation of a particular risk or hazard’ (2001, p. 9).

They argue that the concept of a regime offers four principal benefits in the context of risk regulation. First, because it deals in dimensional systems rather than unified phenomena, it enables the identification and cataloguing of similarities and differences in approaches to regulating risk. Second, by emphasizing the interconnectedness of the different components of the system, it guards against overemphasizing the role of elites. Third, it is able to identify variations in approaches to regulation that are not readily observable through single case studies or broader, macroscopic approaches. Fourth, the concept of a regime is sufficiently flexible to be applied to an entire regulatory system or to a selected component or components. By way of illustration, they suggest that it can be used to study risk regulation across a whole healthcare system or to analyse risk management within a sub‐system – for example, considering the problems posed by ‘dangerous doctors’.

Pollitt *et al.* (2010) use the language of a ‘regime’ to compare the use of performance indicators in the English and Dutch healthcare systems over a 25‐year period. Their analysis shows that broad institutional patterns (particularly cultural and political systems and approaches to organizing and funding healthcare) have a significant influence on the development of performance measurement systems, and are important in explaining stabilities in a system. They suggest that ‘punctuations’ in policy pathways (such as the introduction of national performance indicators) result from a combination of exogenous crises and contingent, opportunistic behaviour by individual actors. However, once performance indicators become embedded in a system, they are sustained by endogenous mechanisms of reproduction which display a ‘logic of escalation’. Performance indicators provide new opportunities for a range of actors to advance their interests, and as a result the ‘systems themselves evolve in a manner that makes it difficult to imagine how they could ever be abandoned’ (p. 26). Pollitt *et al.* conclude that, in conducting a longitudinal analysis of performance regimes, a broad institutional analysis needs to be complemented by particular attention to major changes of course and detailed consideration of the internal logics (and external political effects) of particular policy technologies.

Dean's (1999) analytics of government framework encourages us to pay attention to invisible rationalities that sit behind particular modes of governance. He emphasizes the importance of three elements of a regime: the way in which it problematizes the behaviour which is in need of governance; how the governing activity is achieved (regimes of practice); and the utopian ideal toward which the governing activity is directed. With regard to how the governing activity is achieved, Dean highlights four axes along which the regimes of practice need to be considered: the visibilities created by governance processes and by the use of particular techniques; the knowledge generated by and used within governance processes; the techniques and practices used to achieve the governance (and which may create visibilities, knowledge and identities); and the identities which emerge from and support governance processes.

In this article, we draw upon the frameworks provided by Talbot, Hood *et al.*, Pollitt *et al.* and Dean to analyse three approaches to local government performance assessment introduced in different parts of the UK: Comprehensive Performance Assessment (CPA) in England, Best Value Audits (BVA) in Scotland and the Wales Programme for Improvement (WPI). These ‘whole‐organization assessments’ (WOAs) were at the forefront of an attempt to drive performance improvement in local public services in the UK which has attracted considerable attention from scholars and policy‐makers round the world. Previous research has described, and frequently criticized, deficiencies in the methodologies used in WOAs (Andrews *et al.* 2005; Downe and Martin 2007; McLean *et al.* 2007). There have also been discussions of their impact on local government performance (Downe *et al.* 2010; Nutley *et al.* 2012). But there has been a lack of analysis of how and why they were developed and how they were implemented. This article addresses this gap through a comparative analysis which shows how the concept of a regime can be applied to advance understanding of the origins and operation of approaches to performance assessment, and what shapes the approaches that are taken.

## METHODS AND DATA

We studied the development and implementation of CPA, BVA and the WPI in detail over the period 2002–09 and continued to track subsequent changes in the approaches adopted in all three countries until 2014. Data were gathered from three sources: analysis of policy documents, legislation and statutory and non‐statutory guidance; in‐depth semi‐structured interviews with 45 senior policy‐makers and practitioners from central government, audit bodies, improvement agencies and local government, all of whom had been closely involved in the development or implementation of the performance regimes in their respective countries (see Table 1); and a workshop with a wider group of stakeholders from all three countries to discuss our emerging findings.

**Table 1:** Interviewees

Organizations	Roles
**England (15 interviews)**	
Audit Commission	Chief Executive, Former Chief Executive, Former Head of District Audit
Cabinet Office	Former special adviser to the Prime Minister on health and local government policy
Department of Communities and Local Government	Former Minister of State for Local Government
Department of Constitutional Affairs	Former Parliamentary Under‐Secretary
Improvement & Development Agency	Executive Director, Former Executive Director
Local authorities	Six Chief Executives
Local Government Association	Former Chief Executive
**Scotland (16 interviews)**	
Accounts Commission	Secretary to the Commission, Member
Audit Scotland	Auditor General, Deputy Auditor General, Director of Public Reporting, Assistant Director of Public Reporting
Convention of Scottish Local Authorities	Strategic Director
Improvement Service	Chief Executive
Local authorities	Three Chief Executives and one Assistant Chief Executive
Scottish Government	Best Value Policy Manager, Former Head of Justice Department, Former Minister of Finance, Head of Best Value and Performance
**Wales (14 interviews)**	
Audit Commission in Wales	District Auditor
Local authorities	Three Chief Executives
Wales Audit Office	Performance specialist
Welsh Government	Minister for Social Justice and Local Government, Former Senior Special Adviser to the First Minister, Director of Department of Constitutional Affairs, Equality and Communication, Head of Local Government Policy, Head of Local Government Strategy and Performance, Former Head of Local Government Policy
Welsh Local Government Association	Chief Executive, Performance and Improvement Adviser, Director of Governance and Improvement

We were granted access to all of the main actors (including politicians and officials in central and local government and senior officials from the audit bodies that were responsible for conducting the assessments) in each country. This enabled us to build up a very rich picture of the WOAs and the context within which they operated. Interviewees were identified from the documentary analysis combined with a snowballing approach whereby additional actors were suggested by early interviewees. Interviews covered a range of questions about the approaches to WOAs, how they linked to wider performance improvement initiatives in their country, how they had been developed, what factors shaped their design and implementation and their perceived outcomes. They were taped and transcribed. By triangulating the responses of all interviewees in each country, we were able to construct a rounded picture of how and why the WOAs had been developed and how they operated in practice. We were also able to identify areas of agreement and dissonance between the accounts provided by different actors. Evidence from the documentary analysis and the workshop enabled us to mitigate the risk of elite interviewees exaggerating their own roles in and influence over the design and development of the WOAs.

Our subsequent tracking of approaches to performance assessment at the whole‐authority level since 2009 has drawn on documentary evidence and five knowledge‐exchange events which brought together more than 70 senior policy‐makers, auditors, inspectors and local government representatives from all three countries to analyse and compare developments in local government performance assessment across the UK.

## ANALYSING WHOLE‐ORGANIZATION ASSESSMENTS AS PERFORMANCE REGIMES

Local governments in the UK are responsible for a wide range of services, including education, social services, housing, planning, waste collection and management, leisure and culture, consumer protection and local highways. They also have a role in seeking to promote economic growth and providing community leadership. In Scotland, Wales and most urban areas in England there is a unitary system of local government (i.e. one organization is responsible for all of these services and functions). In some areas in England, responsibility is shared between an upper tier of local government (county councils) and a lower tier (district councils) (Wilson and Game 2006).

In the UK, central government determines what powers are given to local governments and how much they can spend. As a result, policy‐making is highly centralized. National politicians exert considerable influence over local government's spending decisions and take a direct interest in its performance. Historically, the devolved governments in Scotland and Wales have been more inclined than the UK government (responsible for local government in England) to work in partnership with local government (Midwinter and McGarvey 2001; McConnell 2006; Martin *et al.* 2013). Until relatively recently, Wales in particular has adopted a policy of ‘soft steering’ local authorities (Martin and Guarneros‐Meza 2013). This more consensual approach reflects the close‐knit nature of the policy communities in Scotland and Wales and the limited administrative capacity of the devolved governments, which has obliged them to look to local government for policy input (Laffin 2004; Jeffery 2006). In recent years, however, the relationships have become more conflictual and the Scottish and Welsh governments have adopted more hierarchical approaches in their dealings with local government.

Applying the concept of a performance regime to CPA, BVA and the WPI enables us to identify and examine the ways in which interactions between central and local government and other actors determine how WOAs operate and the ways in which they are mediated by formal and informal rules and cultures. In this section we deploy Hood *et al.*'s (2001) framework as the main way of structuring our analysis of the three WOAs. Insights offered by the frameworks developed by Talbot (2010), Pollitt *et al.* (2010) and Dean (1999) are used to supplement this analysis by providing additional interpretations and understandings. This combination of four different – but, we believe, complementary – theoretical perspectives enables us to undertake an analysis which is both cross‐sectional, in that we make comparisons across the three approaches to WOA, and longitudinal, in that we trace developments over time.

### Applying the concept of regimes at different levels of generality: varying institutions and instruments

As explained above, Hood *et al.* (2001) argue that risk regulation regimes can be specified at different levels of breadth or generality. We found that it was possible and indeed necessary to do this because the WOAs in each country were situated within broader approaches to performance assessment and improvement, and as Talbot (2010) suggests, we found that it was helpful to map the key institutions and instruments involved at different levels of regime analysis.

**Figure 1:**
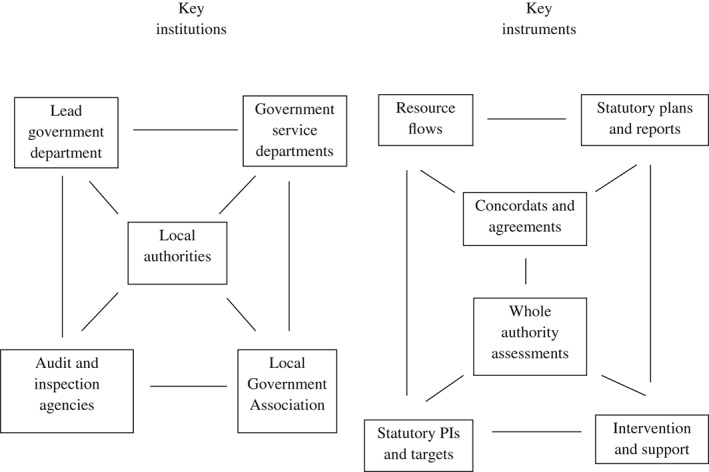
Local government performance improvement regimes in the UK

We identified three ‘nested’ sets of regimes which illustrate how regimes can be narrowly defined or more broadly constructed (May and Jochim 2013). At the ‘lowest’ level were WOAs. These operated as bounded systems that could be analysed in their own right. However, their boundaries were permeable, since WOAs were part of a ‘second level’ of broader improvement regimes which consisted of a wider range of instruments (including government grants, reporting requirements, concordats and contracts, and statutory performance indicators) that were intended to work together to promote improvements in local government performance (figure 1). We found that there were interactions between the WOAs and this wider array of instruments. For example, the criteria used to assess local government performance in CPAs in England were strongly influenced by the priorities and performance targets set out in Local Area Agreements made between central government departments and individual local governments. In Scotland, assessors used statutory performance indicators to inform BVAs. In all three countries, critical WOA reports could trigger external support, including the insertion of interim management teams to try to ‘turn around’ the performance of ‘failing councils’.

These second‐level performance improvement regimes were themselves situated within a higher (third) level of public service reform strategies which were developed by the three national governments in conjunction with a range of other institutional actors. For example, the use of ‘star ratings’ as a means of identifying and incentivizing improvement in local government in England reflected the UK government's belief in the value of creating competition between local governments and was echoed in the use of the same instruments in a range of public service delivery settings, including hospitals. By contrast, until 2011 the Welsh government's public service reform agenda explicitly rejected the use of star ratings (and ‘naming and shaming’) and the WPI was in line with this approach (Bevan and Wilson 2013).

We found that the key institutions involved and the influence they wielded varied between these three different levels of analysis. For example, WOAs were largely shaped by three key sets of actors: the central government department with responsibility for local government policy, the principal local government audit body and the local government associations (bodies which represent and promote the collective interests of local governments). At this level of analysis, many of the institutions that Talbot (2010) suggests might make up a performance regime, including other central government departments, service‐specific inspectorates (such as the inspectorates for schools), professional institutes, business interests and the voluntary sector had little discernible influence, and the legislatures and judiciary also had only minor roles. However, at the second level, approaches to local government performance improvement involved a wider range of central government departments including those with responsibility for finance, education, environment, transport and health. At the third level of public services reform strategies (which is the level at which Talbot applied the concept of a performance regime), a wider set of actors came into play, including business interests, public service user groups and public policy think‐tanks. Audit bodies and local government associations exerted much less influence than at the WOA level.

So, we found that Talbot's description of a performance regime provided a helpful framework for identifying the key institutions and instruments involved in promoting performance improvement in local government at different levels. Applying the framework at different levels helped to remind us of the need not just to analyse the interactions among institutions at the level of WOAs, but also to take account of the interactions between WOAs and the higher‐level local government performance improvement systems and public service reform strategies within which they are embedded. But mapping institutions and instruments was not sufficient, because it did not identify or account for the differences between England, Scotland and Wales in the ways in which WOAs were developed and implemented. The same institutions and instruments shown in figure 1 were involved in all three countries, but differences in the ways in which they interacted gave each of the regimes a distinct ‘feel’. To understand this we needed to analyse the origins and operation of the WOAs in detail, and the concept of a regime articulated by Hood *et al.* (2001) provided the most useful framework for doing this.

### Regime context, content and control

Hood *et al.* (2001) argue that the context and content of a regime shape, and in turn are shaped by, the way in which control is exercised. So our analysis of CPA, BVA and the WPI considers these three dimensions of the WOAs in turn.

Hood *et al.* (2001) identify three important features of the context within which risk regulation regimes operate: the type of risk which they regulate; public attitudes toward the risk; and the nature of organized interests which have a stake in its regulation. We found that all three of these elements proved important to understanding the three WOAs and the differences between them. The type of risk that WOAs were intended to regulate was a complex and contested issue. Some interviewees (particularly those working in local government) defined WOAs as a way of identifying the relatively small minority of local governments that were failing to meet basic standards of service. Others (mostly in central government) thought that they targeted a much broader spectrum, including what they described as ‘coasting councils’ (a relatively large number of organizations whose performance was near the average and which central government believed should be aspiring to do much better).

Our analysis suggested that public preferences and attitudes did not influence WOAs directly. The performance of local government mattered to central government ministers but interviewees believed it had low public salience, partly because few citizens understand which services UK local government provides (Ipsos MORI 2004). There was no direct public involvement in the process of assessing performance, although some interviewees believed that the results of WOAs would empower citizens to hold their local governments to account. The architects of CPA in England insisted that publishing results in the form of ‘star ratings’ would increase transparency and spark public interest in performance (Audit Commission 2002). CPA results for all upper tier and unitary local governments were published every year and on the same day to encourage comparisons between them, and there is evidence that this had an impact on voting intentions where local governments received poor ratings (James and John 2007). In Scotland, BVA reports were published, but not on the same day, and they did not give local governments an overall score, which meant that the ‘headline message’ was much less clear‐cut than in England. In Wales, WPI reports were not published at all, nor were they seen by central government. The audit body produced an annual report of its overall findings but this contained no information about individual local governments. This level of secrecy meant that the public had no way of knowing how well their local government was performing and became a source of increasing frustration to ministers who were unable to use WPI reports to monitor each organization's progress.

As suggested by Hood *et al.* (2001), the nature of the organized interests also emerged as an important factor in shaping the three regimes we studied, and it was key to understanding variations between them. As noted above, the same institutions – government departments, audit bodies and local government associations – were involved in all three countries. However, differences in central–local relations and the distribution of power among the institutions led to significant differences between the WOAs. In England, CPAs were designed by senior Audit Commission officials and imposed top‐down. The Audit Commission reported to senior civil servants and special advisers in the (then) Department of Transport, Local Government and the Regions. The Local Government Association was not closely involved and although a small group of local governments were invited to pilot the process, this did not result in any fundamental change to the Audit Commission's original proposals.

In Scotland, the process for designing BVAs was much more inclusive and consensual, with local government enjoying far greater influence. A task force with representation from the Scottish government, Audit Scotland, local government, trades unions, professional organizations and the Scottish Consumer Council designed the regime and as a result it enjoyed a high level of support. In Wales, the WPI was developed by a tripartite group of senior civil servants, Audit Commission officials and the Welsh Local Government Association. Local government had strong support from the minister and exerted considerable influence. The auditors found themselves in a much weaker position than their counterparts in England or Scotland and were handed a regime that they believed had been captured by producer interests and lacked sufficient rigour and transparency.

Turning to regime content, Hood *et al.* (2001) identify three elements – size, structure and style. Size refers to how much regulation is brought to bear on a risk. Structure refers to the way in which regulation is organized (the distribution of regulatory costs, the complexity of the system and interfaces with other regimes). Style refers to the conventions and attitudes of those operating a regime, including the degree of ‘regulatory zeal’ they bring and the formal and informal processes through which regulation operates. Like the concept of context, these proved useful in our analysis, although not all were equally enlightening.

Hood *et al.* (2001) identify two measures of regime size – ‘policy aggression’ and the level of investment in a regime. We found variations in ‘aggression’, which in turn reflected the differences in policy and in central–local relations, as well as beliefs about the best way to motivate local governments to improve their performance (what we have described elsewhere as ‘theories of improvement’ (Downe *et al.* 2010)). CPAs were perceived by interviewees to be very aggressive. The ‘naming and shaming’ of ‘poor’ performers was, they said, intended to provide a ‘shock to the system’. The Audit Commission also invested heavily in CPA. As noted above, it conducted annual assessments of all 151 upper tier and unitary local governments in England, plus less frequent CPAs in the 201 district (lower tier) councils. CPAs cost £28m per annum to administer (House of Commons 2013) and also consumed significant amounts of local government time in preparing the documents and performance data required by assessors, hosting visits by them and responding to reports. The estimated costs of compliance vary between £25m and £45m annually (OPM 2010).

The Audit Commission also invested a great deal of its own political capital in CPA. According to the senior Audit Commission officials who designed it, it was seen as a way of restoring the audit body's reputation with ministers following what was seen as the botched implementation of the ‘Best Value’ inspections which preceded it. It proved spectacularly successful in this regard, and between 2003 and 2008 the Audit Commission enjoyed a period of unprecedented expansion and influence with central government. However, growing resentment toward CPA among local politicians eventually contributed to the Audit Commission's demise when there was a change of central government in 2010 and ministers declared that the audit body had ‘lost its way’ and must be abolished (Shapps 2011).

The WPI was also seen as time‐consuming and resource‐intensive but was less onerous than CPA. As one interviewee explained, it was felt that there was no need in a small country for ‘an over‐engineered system like CPA’. Each local government was required to prepare a self‐assessment each year. In parallel, auditors conducted their own ‘risk assessments’ and the two parties then agreed improvement plans. In contrast to the ‘external shock’ provided by CPA, the aim was that local governments would identify and take responsibility for addressing their weaknesses since, as an interviewee from the Welsh government explained, ‘change which is owned by local government is more sustainable and ultimately more effective’. Because WPI reports on individual local governments were unpublished, the WPI was not perceived as aggressive or intrusive. One local government chief executive explained that ‘performance and improvement dominate my life but the Wales Programme for Improvement doesn't’.

BVA was perceived to be far less aggressive than CPA, but more challenging than the WPI. According to one interviewee, ‘you don't need strong arm tactics … [because] the community is close enough that word gets round quickly’. Scale was also important because, according to another interviewee, with just 32 local government units in Scotland, ‘we can capture what is truly happening in local councils through this kind of [flexible] methodology’. Because BVAs were conducted on a three‐year cycle, they required less investment (by the auditors and local governments) than their English or Welsh counterparts.

The structural dimensions of the three regimes were not a significant distinguishing feature, but were important in explaining change over time. The distribution of costs between regulators and regulatees was similar in all three countries. In all three cases, assessors drew on information from self‐assessments but used a variety of evidence to supplement this. Local governments everywhere complained of the burden of compliance costs and a lack of ‘joining up’ with service‐specific inspectorates which led to multiple requests for similar information. From 2009 onwards, the complexity of the system, particularly the interfaces with other performance assessments, became a distinguishing feature of the ways in which the regimes developed. CPA was able to operate in relative isolation from other performance assessment and service‐specific inspection regimes (mainly using them as sources of data for input into the CPA process), but CPA was replaced in 2009 by Comprehensive Area Assessments (CAA), which required more collaborative working across inspection agencies.

Similarly, developments in the Scottish BVA regime after 2009 included a new system of shared risk assessments, which require Audit Scotland to work together with other inspectorates to agree on the key risks in each local government and to coordinate an inspection plan to address these risks. In Wales, the Local Government Measure (2009) made significant changes to the WPI. The focus shifted from risk assessment to securing continuous improvement, efforts were made to improve coordination between inspectorates and local governments were required to release performance data to the public.

In terms of style, all three regimes exhibited a high degree of rule orientation which was designed to ensure transparency about methods and consistency of approach in the assessment of local government. All three were based on the assumption that ‘corporate capacity’ is vital to improvement. They therefore sought to test the effectiveness of a local government's political and managerial leadership, its clarity of purpose, its use of resources, and performance management systems. CPA assessors used a standardized methodology and scoring system based on ‘key lines of enquiry’ which focused on these issues as well as the performance of key services delivered by local governments. BVAs and WPIs adopted more flexible approaches designed to respond to variations in local context and the priorities defined by each local government. However, they used similar judgement criteria to CPA, and interviewees from Scotland reported that in practice the process was not as customized or as useful as they hoped. BVA reports are about ‘the weakness of corporate governance first and foremost’, said one local government interviewee, while another suggested that ‘they are like a report card on the chief executive’.

The level of ‘regulatory zeal’ varied between the regimes. Auditors in England and Scotland were strongly committed to CPAs and BVAs, respectively; their Welsh counterparts were less convinced about the value of the WPI. This reflected differences in central–local government relations described above, and in particular the close relationship between ministers and the Welsh Local Government Association. One interviewee admitted that ‘probably too much was done by conversations between me and XXX … I always felt really uncomfortable that we weren't doing all of that stuff formally’.

As explained above, in addition to context and content, Hood *et al.* (2001) emphasize the importance of control mechanisms in shaping a regime. They identify three types of controls – the ways in which standards are set (directors), how information is gathered to check if standards are being met (detectors) and how behaviour is modified to ensure compliance with the standards (effectors). The key control mechanisms in each of the three WOAs are summarized in table 2.

**Table 2:** The exercise of control within CPA, BVA and WPI

	Standard setting	Information gathering	Behaviour modification
	‘Directors’	‘Detectors’	‘Effectors’
England: Comprehensive Performance Assessments (CPA)	Uniform rules‐based scoring system reflecting national priorities.	An element of self‐assessment but largely auditor‐led using key lines of enquiry.	Naming and shaming – overall scores published alongside narrative reports.
			Staged escalation of sanctions and interventions.
	Criteria for assessing service performance tightly defined.		
			Some peer and capacity‐building support for improvement efforts.
	Corporate assessment criteria more impressionistic.		
Scotland: Best Value Audits (BVA)	Standards articulated at level of principles.	Initial self‐assessment, but then auditor‐led.	Narrative BVA reports placed in public domain.
	Tailored application depending on local priorities.	No scoring of performance against standards.	
			Some sanctions for poor performance but treated as a last resort.
			Focus on education and persuasion through agreeing an improvement plan.
Wales: Wales Programme for Improvement (WPI)	Principle‐based standards.	Emphasis on self‐assessment.	Joint risk assessments confidential to auditors and each local council.
	Tailored to each council through joint risk assessment process (auditors working with each council).	No scoring of performance against standards.	
			Jointly agreed improvement plan.
			Emphasis on self‐directed improvement.
			Intervention as a last resort.

CPA exercised control through setting explicit, rules‐based standards that were designed to be applied in a fairly uniform manner to all local governments. There was an element of self‐assessment but, in practice, information gathering was largely auditor‐led. Performance scores were published in league tables and behaviour modification mechanisms emphasized naming and shaming. This was complemented by a staged escalation of sanctions and interventions combined with resources for capacity‐building and peer support.

By contrast, in Wales, performance standards were influenced by broad principles rather than specific rules and were applied selectively on the basis of a risk assessment undertaken by auditors in conjunction with each local government. Self‐assessment played a much stronger role in the process of information gathering and this was reflected in the approach to behaviour modification, which emphasized self‐directed improvement.

The approach to control in the BVA regime can be characterized as sitting between the standardized, rule‐based and punitive approach of CPA and the customized, joint assessment and improvement approach of the WPI. BVA standards were articulated as principles to be applied in a way which recognized the particular circumstances in each locality. Although information gathering began with a self‐assessment process, it was largely led by auditors. BVA reports were placed in the public domain, but the bespoke nature of each report muted this ‘effector’ and in practice more emphasis was placed on informal negotiation behind the scenes, which resulted in the development of an improvement plan agreed between the auditors and local government.

In summary, the framework developed by Hood *et al.* (2001) proved very useful as a means of documenting the key characteristics of WOAs, comparing them and understanding the reasons for the differences that we observed. However, we found that the regimes evolved over time. This raised the question of whether applying a regimes perspective can assist longitudinal as well as comparative analysis.

### Understanding change over time

Pollitt *et al.* (2010) argue that performance regimes are relatively stable but are subject to development and change over time due to endogenous growth and exogenous shocks. Thus cross‐sectional analysis cannot tell the whole story of a regime; a temporal dimension is also important. Our analysis bears this out, as all three WOAs developed over time and we found that changes were driven by a combination of endogenous and exogenous factors.

CPA and BVA became more formalized and sophisticated. There were changes in the criteria they used (the key lines of enquiry), in resource requirements and in reporting arrangements. Interviewees attributed these to the need for audit bodies to respond to external challenges to the validity and reliability of the regimes. As predicted by Pollitt *et al.*, they also exhibited an internal logic of escalation. CPA was first applied to unitary and upper tier local governments but then extended to the lower tier districts. The performance criteria used in assessments were expanded with the introduction in 2005 of a so‐called ‘harder test’ and the introduction of CAA in 2008 which placed increased emphasis on the need for public services to work in partnership. But the biggest exogenous shock to the system in England was the election of the Conservative‐led coalition government in 2010, which led to the abolition of the Audit Commission and the introduction of a process of voluntary assessment known as Corporate Peer Challenge in place of mandatory external assessment by the audit body.

Developments in the BVA regime in Scotland after 2009 were also shaped by changes in the broader public services reform strategy taken by central government. As in England, the Scottish government placed greater emphasis on partnership working. Community Planning Partnerships (involving health, the police and other agencies) were given a central role in the delivery of public services and this meant that Audit Scotland had to adapt BVAs to assess their performance rather than focusing solely on local government. As described above, the way in which the WPI in Wales operated was refined in 2005 and then again in 2009. Here too there have been major changes in the broader performance assessment regime as a result of external shocks. The publication of PISA (Programme for International Student Assessment) scores indicating that educational attainment in Wales lags behind the rest of the UK led to the introduction of ‘banding’ of schools on the basis of external education inspection scores, and serious failures in other local services triggered external intervention in a number of local governments and an independent review of public service governance and delivery (Welsh Government 2014).

## DISCUSSION AND CONCLUSION

Having applied the concept of a regime to the analysis of approaches to UK local government performance assessment, we now return to the four specific questions raised at the beginning of this article.

### Is a regimes perspective a useful way of identifying and analysing differences between approaches to performance assessment in public services?

Our analysis supports the claim of May and Jochim (2013, p. 42) that ‘the regime lens helps to illuminate the realities of how a given set of problems is addressed and the political dynamics that are engendered by those realities’. The ‘risk regulation’ and ‘institutions and instruments’ frameworks offered by Hood *et al.* (2001) and Talbot (2010) are both helpful in characterizing the contrasting approaches to performance assessment in England, Scotland and Wales. We find the more detailed parameters of the risk regulation framework to be the most useful when it comes to analysing differences between the three regimes at the WOA level, while the institutions and instruments perspective is important in helping to maintain a sense of the wider regime.

Our analysis also demonstrates the importance of drawing on a range of evidence in investigating the various dimensions of the risk regulation framework. The high‐level policy documents that we analysed emphasized the differences in approach in the three countries, partly because of a desire by devolved administrations to differentiate their policies from those of the UK government operating in England (Nutley *et al.* 2012). Audit manuals focus on the formal rules, practices, tools and techniques associated with a regime, which appeared to be similar across all three countries. Interviews gave insights into the informal (sometimes hidden) processes which lay behind regimes, which meant that they ‘felt’ very different ‘on the ground’. As an interviewee in Scotland explained, ‘although many aspects of public service audit and inspection in England and Scotland look as if they are converging, the overall feel of these regimes … is different’.

### At what level of analysis is the concept of a performance regime most useful?

Our analysis demonstrates that the concept of a regime can be applied at different levels of analysis. We have examined how a single policy initiative (WOAs) operates at a national level (across all local governments). This is distinct from a more micro‐level analysis of how WOAs play out in particular local governments, a meso‐level approach which focuses on all of the instruments which make up a local government performance regime or a macro‐level approach that investigates an entire public service reform strategy. Analysis of specific local governments would have the advantage of offering a detailed and nuanced account of how a regime is implemented in a particular context, but raises questions about the external validity of a small number of cases. Meso‐ and macro‐level analyses are good at highlighting the broader context with in which specific policy initiatives operate. However, they run the risk of focusing on policy intentions rather than how policies are implemented on the ground. We suggest that a regimes perspective can be used at all of these levels, but that the analysis of a specific instrument at national level has an important contribution to make to understanding its origins and operation. It could be enriched by drawing on data from studies conducted at micro, meso and macro levels.

### Can the concept of a performance regime help to explain differences in approaches to performance assessment between jurisdictions and over time?

Although regimes are conceptualized as relatively stable systems, our study shows that a regime analysis should not be limited to characterizing how the system works at a particular point in time. Hood *et al.* (2001) point out that it is the ‘relationship profile’ between the elements in the regime that is important, and our analysis shows that these are not static relationships. Examining the interactions between the various institutions and instruments involved in a regime sheds light on the way it changes over time and why. Pollitt *et al.* (2010) are right to also draw attention to the internal logics of escalation embedded within a regime and the way that significant shifts in a regime are caused by exogenous shocks. This is in line with Hood *et al.*'s conclusion that it is context rather than the inner lives of regimes which explains much of their variety. A regime approach can shed some light on the relative strength of various interest groups and institutions and this helps to explain some of the system dynamics. But it does not address deeper questions about what drives adoption of performance regimes or whose interests they serve. These issues could be addressed by drawing on Dean's (1999) analytics of government framework, which helps to focus attention on questions of power, privilege and control and could help to uncover the invisible rationalities for introducing performance regimes which are belied by the managerialist rhetoric usually employed by policy‐makers and auditors.

### How might the concept of a performance regime be developed in the future to advance our understanding of approaches to performance assessment?

Given that different approaches to performance assessment and improvement are often described as regimes, it is important to consider what this means in analytical terms. We have argued that identifying the key dimensions of a performance regime and using these as an analytical framework provides a more robust basis for comparing different approaches to performance management than merely describing them in broad‐brush terms. We have demonstrated the appropriateness of Hood *et al.*'s risk regulation framework as a starting point for this dimensional analysis. We have also shown how Talbot's institutions and instruments framework complements this analysis and can be applied to regimes which operate at different ‘levels’, including individual WOAs, the performance improvement systems of which WOAs are part and broader public service reform strategies.

Our analysis has also considered how and why regimes change over time. Hood *et al.*'s and Talbot's frameworks can help to map changes, but to understand the drivers of change we need to draw on other frameworks. Pollitt *et al.*'s analysis of endogenous and exogenous factors (internal logics and external shocks) is a starting point, but there is a need to develop a richer understanding of system dynamics as part of regime analysis. There are benefits in combining different frameworks in this way in order to develop a more rounded perspective of a regime. But it is important to guard against the danger of reification. In the final analysis, regimes are analytical constructs rather than directly observable entities. Moreover, there are two gaps in our analysis that we believe future research might usefully seek to address.

First, we have not sought to evaluate whether WOAs were effective in driving improved performance in local government. None of the theoretical frameworks on which we drew were concerned with the impacts of a regime, and as we have discussed elsewhere, there are formidable measurement and attribution problems associated with attempts to assess the impact of CPA, BVA and WPI (Martin *et al.* 2010). Nonetheless, a regime analysis has the potential to inform a theory‐based or realist evaluation of their impacts because it can unpack how WOAs operate: the theories of improvement which underpin them, the different contexts in which they operate and the control mechanisms which they use. A regimes perspective could also help to identify misalignments in design or implementation which could undermine a regime's legitimacy, coherence and durability (May and Jochim 2013) – for example, a mismatch between policy ambitions and the available political, financial and/or technical resources.

Second, our analysis has focused primarily on the surface features of the three performance regimes we have studied. We have not blindly accepted the rhetoric associated with these regimes or relied solely on normative descriptions of their operation. So, in a sense, we have scratched the surface to consider how they operated in practice. However, we have not sought to uncover the invisible rationalities that sit behind these regimes, as would be suggested by Dean's (1999) analytics of government framework, although our analysis does point to their existence. For example, we found that all three performance regimes were underpinned by largely unspoken theories of improvement and that they were refined and, in the case of CPA, replaced as successive governments and ministers sought to make their own mark.

In conclusion, we believe that applying the concept of performance regimes adds new insights but also has limitations. Any framework privileges some questions, bringing some issues to the fore and pushing others into the background. In particular, we have found that while studying the constituent parts of a performance improvement regime is extremely valuable, it does not necessarily capture the essence of its operation. Nor does it tell us much about the underlying forces which have led to the growth of particular approaches to performance assessments and their trajectories over time. Answers to these questions may require the application of different theoretical perspectives. In particular, we believe there could be merit in adopting a dual perspective that combines mainstream regimes analysis (of the type provided in this article) with the kind of discourse analysis which has been advocated by Dean.
